# The Redox Role of G6PD in Cell Growth, Cell Death, and Cancer

**DOI:** 10.3390/cells8091055

**Published:** 2019-09-08

**Authors:** Hung-Chi Yang, Yi-Hsuan Wu, Wei-Chen Yen, Hui-Ya Liu, Tsong-Long Hwang, Arnold Stern, Daniel Tsun-Yee Chiu

**Affiliations:** 1 Department of Medical Laboratory Science and Biotechnology, Yuanpei University of Medical Technology, Hsinchu 30041, Taiwan; 2 Research Center for Chinese Herbal Medicine, College of Human Ecology, Chang Gung University of Science and Technology, Taoyuan 33043, Taiwan; yhwu03@mail.cgust.edu.tw; 3 Graduate Institute of Biomedical Sciences, College of Medicine, Chang Gung University, Taoyuan 33043, Taiwan; d56610@yahoo.com.tw; 4 Department of Medical Biotechnology and Laboratory Sciences, College of Medicine, Chang Gung University, Taoyuan 33043, Taiwan; liuhy@mail.cgu.edu.tw; 5 Research Center for Food and Cosmetic Safety, College of Human Ecology, Chang Gung University of Science and Technology, Taoyuan 33043, Taiwan; htl@mail.cgu.edu.tw; 6 Graduate Institute of Natural Products, College of Medicine, Chang Gung University, Taoyuan 33043, Taiwan; 7 Chinese Herbal Medicine Research Team, Healthy Aging Research Center, Chang Gung University, Taoyuan 33043, Taiwan; 8 Department of Anaesthesiology, Chang Gung Memorial Hospital, Taoyuan 33043, Taiwan; 9 Department of Chemical Engineering, Ming Chi University of Technology, New Taipei City 24301, Taiwan; 10 Research Center for Chinese Herbal Medicine, Graduate Institute of Health Industry Technology, College of Human Ecology, Chang Gung University of Science and Technology, Taoyuan 33043, Taiwan; 11 School of Medicine, New York University, New York, NY 10016, USA; stern@nyulangone.org; 12 Department of Pediatric Hematology/Oncology, Linkou Chang Gung Memorial Hospital, Taoyuan 33043, Taiwan; 13 Healthy Aging Research Center, Chang Gung University, Taoyuan 33043, Taiwan

**Keywords:** G6PD, redox signaling, cell growth, cell death, cancer

## Abstract

The generation of reducing equivalent NADPH via glucose-6-phosphate dehydrogenase (G6PD) is critical for the maintenance of redox homeostasis and reductive biosynthesis in cells. NADPH also plays key roles in cellular processes mediated by redox signaling. Insufficient G6PD activity predisposes cells to growth retardation and demise. Severely lacking G6PD impairs embryonic development and delays organismal growth. Altered G6PD activity is associated with pathophysiology, such as autophagy, insulin resistance, infection, inflammation, as well as diabetes and hypertension. Aberrant activation of G6PD leads to enhanced cell proliferation and adaptation in many types of cancers. The present review aims to update the existing knowledge concerning G6PD and emphasizes how G6PD modulates redox signaling and affects cell survival and demise, particularly in diseases such as cancer. Exploiting G6PD as a potential drug target against cancer is also discussed.

## 1. Introduction

The central roles of glucose-6-phosphate dehydrogenase (G6PD) are the production of ribose and the reducing equivalent nicotinamide adenine dinucleotide phosphate (NADPH) via the pentose phosphate pathway (PPP). Both products are vital for the synthesis of many biological building blocks, such as nucleic and fatty acids. It has long been known that NADPH is extremely important in the maintenance of antioxidant defenses [1]. A preponderance of evidence has emerged recently to indicate that NADPH also serves as a pro-oxidant to generate reactive oxygen species (ROS) and reactive nitrogen species (RNS) as signal molecules for promoting cellular processes, such as cell growth. Clinically, G6PD deficiency is the most pervasive X-linked enzymopathy in the world. G6PD-deficient individuals tend to suffer from red cell disorders, including jaundice and drug- or infection-induced hemolytic anemia. These disorders are mostly due to a point mutation in G6PD [2]. Severe G6PD deficiency is intolerant for growth and development in animal models [3,4,5,6,7,8], while a modest increase of G6PD promotes a healthy lifespan [9].

Many excellent reviews have discussed the pro-survival role of G6PD [10,11,12,13,14,15]. How G6PD as a part of PPP affects cells, including cancer cell growth and death, has not been clearly defined. G6PD enhances tumor growth by maintaining intracellular redox homeostasis [16]. G6PD activity is increased in several types of cancers, including bladder, breast, endometrial, esophageal, prostate, gastric, renal, hepatic, colorectal, cervical, lung, and ovarian cancers, glioblastomas and leukemia, as well as gliomas [17,18,19,20,21,22,23,24,25,26,27,28,29,30,31,32,33,34,35,36,37,38,39,40,41,42,43,44,45,46,47,48,49,50,51,52,53,54,55,56,57,58]. The current review provides an update of the existing knowledge concerning G6PD and focuses on how G6PD is involved in redox signaling and how it affects cell survival and death, particularly in diseases such as cancer. Exploiting G6PD as a potential drug target against cancer is also discussed.

## 2. G6PD and Cellular Signaling with Emphasis on Redox Signaling

### 2.1. The Relationship between G6PD and Reactive Species (RS)

The production of superoxide by NADPH oxidase (NOX) and nitric oxide (NO) by NO synthase (NOS) is NADPH-dependent [59]. PPP is the major pathway for NADPH generation. Oxidative stress is considered a risk factor for aging and chronic diseases [60,61]. Low molecular weight signaling molecules play an important role in human health and disease. They are highly reactive and easily diffusible molecules that include ROS, RNS, reactive sulfur species (RSS), carbon monoxide, ammonia, and methane [59,62,63,64,65]. Questions of whether or not G6PD status affects the production of ROS, RNS, and RSS and how G6PD regulates the downstream redox signaling pathways, as well as its impact on human health and diseases, are of great interest.

Intracellular RS production is regulated by enzymatic reactions, which can subsequently affect the function and structure of proteins as well as the transcription of genes by modification of cysteines [66,67]. However, excess RS also contributes to the development of chronic diseases by attacking cellular components, such as proteins, lipids, and nucleic acids, leading to cellular dysfunction [68]. NO is a radical as well as an effector and messenger. Interaction between NO and ROS generates RNS. Both ROS and RNS can react with cysteine thiols to form RSS [69]. Hydrogen sulfide (H_2_S) has been initially considered as an environmental toxin through inhibition of mitochondrial respiration [62]. Endogenous H_2_S plays a role in diverse biochemical pathways governing signal transduction, bioenergetics, and lifespan [63,70]. Bacterial H_2_S is considered as a protective factor conferring antibiotic resistance and is involved in the host immune response [64]. The inhalation of H_2_S by mice causes hibernation-like behavior associated with reduced body temperature and metabolism [71].

Due to the complex interaction of signaling molecules and downstream effectors, the reactive species interactome (RSI) has been introduced as an integrative concept to delineate the complexity of the multiple level redox regulation system [65]. In response to various stress and environmental cues, the RSI increases fitness and flexibility at cell, tissue, and organismal level through rapid sensing and adjustment. Full understanding of the mechanistic action of the RSI opens the opportunity to appreciate redox biology in human health and disease as well as providing novel strategies of prevention or intervention for precision medicine.

### 2.2. The Interaction between G6PD Status and Reactive Species

Human G6PD-deficient granulocytes exhibit impairment of hydrogen peroxide and superoxide production [72]. Similar to the finding in cells, lower superoxide release, and reduced atherosclerotic lesions have been observed in G6PD-deficient mice crossbred with ApoE null hemizygous mice [73]. G6PD-derived NADPH is responsible for superoxide production as found in a pacing-induced heart failure canine model [74]. The ventricular tissue homogenates show an increase in NADPH, superoxide, and G6PD activity. Treatment with a NOX inhibitor gp91(ds-tat) or a G6PD inhibitor, 6-aminonicotinamide (6-AN), significantly reduces superoxide generation in the failing heart homogenates. The upregulation of myocardial G6PD provides sufficient NADPH and fuels the superoxide-producing enzymes, suggesting a redox role for G6PD in the pathogenesis of heart disease [74]. G6PD regulates nuclear superoxide production by cooperating with NOX4 in the hepatocytes, where G6PD and NOX4 are co-localized in the nucleus [75]. The close relationship between G6PD and NOX4 maintains ROS homeostasis and promotes downstream redox signaling, including STAT3, c-SRC and SHP2, in melanoma cells [16]. Overexpression of G6PD in bovine aortic endothelial cells (BAECs) diminishes ROS accumulation following exposure to hydrogen peroxide, TNF-α or xanthine oxidase. Upregulation of G6PD in BAECs maintains the reduced form of glutathione [76].

NO generation can be stimulated by cytokines and NO donors [77,78]. NO affects cell survival, the immune response, insulin signaling, and stress disorders and provides vascular and neural protection [72,76,79,80,81,82,83,84]. NO production is dependent on G6PD status [10]. Increased levels of NO and G6PD have been found in the saliva of refugees suffering from stress and anxiety [80]. Upon lipopolysaccharide (LPS) or 12-myristate 13-acetate (PMA) stimulation, human granulocytes produce nitrite (derived from NO). Human granulocytes lacking G6PD fail to generate NO in the presence of LPS or PMA [72]. The cytokine IL-1β enhances inducible nitric oxide synthase (iNOS) expression and NO production in pancreatic islet cells causing cell death and disruption of insulin secretion. IL-1β upregulates G6PD activity and reduces cyclic adenosine monophosphate (cAMP) levels. 8-bromo-cAMP, an activator of cAMP-dependent protein kinase, increases G6PD activity, while a protein kinase A (PKA) inhibitor decreases G6PD activity [79].

G6PD status is positively correlated with NO production. Suppression of G6PD by the biochemical inhibitor dehydroepiandrosterone (DHEA) or an antisense oligonucleotide reduces the IL-1β-induced NO level, indicating that cAMP-dependent PKA enhances G6PD status stimulated by IL-1β-derived NO. Sodium nitroprusside, an NO donor, stimulates cell growth in G6PD-normal fibroblasts but induces apoptosis in G6PD-deficient fibroblasts [81]. Treatment with Trolox, an antioxidant, or ectopic expression of G6PD reverses NO-induced apoptosis in G6PD-deficient fibroblasts, suggesting a pro-survival role for G6PD. Reduced G6PD activity in endothelial cells is associated with elevated ROS and decreased NO bioavailability [82]. Overexpression of G6PD in BAECs treated with bradykinin also enhances cGMP and NOS activity. This results in an increase in bioavailable NO [76].

NO availability is essential for the regulation of leukocyte adhesion in the endothelium [85]. G6PD-deficient endothelial cells display lower level of endothelial nitric oxide synthase (eNOS), NO, and glutathione (GSH). Treatment of G6PD-deficient endothelial cells with high concentrations of glucose as a pro-atherosclerotic stimulus upregulates ICAM-1 and VCAM-1, as well as the oxidant markers, ROS, NOX4, and iNOS. By contrast, l-cysteine (a GSH precursor) attenuates these oxidative markers, suggesting that G6PD and GSH play a role in endothelial cell protection associated with NO availability [85]. LPS increases the mRNA expression of G6PD and glucose utilization of the PPP independent of iNOS in cultured rat astrocytes, while inhibition of NF-κB blocks the expression of G6PD and iNOS [83]. Inhibition of G6PD in rat astrocytes by DHEA prevents PPP activity and lowers NADPH and the GSH/ glutathione disulfide (GSSG) ratio. The alteration of the GSH/GSSG ratio due to DHEA can be reversed by an iNOS inhibitor (AMT). These observations indicate that G6PD protects astrocytes from NO-mediated cell damage.

Peroxynitrite is an NO-derived neurotoxin [86,87]. It rapidly increases the activity of the PPP in neurons and astrocytes in primary culture, which leads to an increase in NADPH [84]. NO causes glutathione oxidation, NADPH consumption, and apoptosis in neurons but not astrocytes. Peroxynitrite treatment can counteract the effect caused by NO in neurons. Both endogenous and exogenous peroxynitrite induces G6PD activity in PC12 cells. Overexpression of G6PD confers resistance to NO-mediated apoptosis, while G6PD knockdown exacerbates cellular injury. Taken together, the cross-talk between G6PD and NO is crucial for cell protection.

H_2_S, an endogenous gasotransmitter, is involved in many biological functions, including neuronal regulation [88], smooth muscle relaxation [89], vascular relaxation and blood pressure regulation [90], inflammation [91,92], cell death signaling [93], and metabolism [71,94,95]. Overstimulation of the β-adrenergic receptor (β-AR) by isoproterenol in hypertrophic cardiomyocytes rapidly reduces the endogenous H_2_S level. Treatment with the H_2_S agonist (NaHS or norepinephrine) to augment H_2_S production suppresses the hypertrophy stimulated by the β-AR in cardiomyocytes [96]. Rats with transverse aortic constriction have approximately half the H_2_S level compared to normal rats [96]. Treatment of NaHS enhances G6PD activity, while the G6PD inhibitor (6-AN or DHEA) decreases hypertrophic responses in cardiomyocytes. β-AR upregulates G6PD expression and activity in rats [96]. β-AR reduces cardiac p53, which negatively regulates G6PD by preventing G6PD dimerization [48]. G6PD inhibitors (either 6-AN or DHEA) reverse the β-AR-induced effect in rats with cardiac hypertrophy. Enhancing G6PD activity directly or inhibiting G6PD activity by p53 indicates that G6PD plays a critical role in mediating cardiac function regulated by H_2_S. Global transcriptome analysis reveals that H_2_S modulates an integrated metabolic network regulating cellular redox homeostasis. Consistent with these findings, several common biological processes emerging from the transcriptome data show that G6PD is a critical node modulating the effects among metabolic processes downstream of H_2_S [96].

Carbon monoxide (CO) is also a gaseous signaling molecule produced in humans. The major roles of CO are the modulation of the cardiovascular system, inhibition of platelet aggregation and adhesion, and neuronal development [97]. Like NO and H_2_S, CO is antiapoptotic, anti-inflammatory, and vasodilatory. It also promotes vascular growth [98,99,100]. The abnormal metabolism of CO has been associated with diseases, including heart failure, hypertension, inflammation, and neurodegeneration [101,102]. Since CO is produced from hemoglobin by heme oxygenase 1 and 2, it can be used as an index of heme catabolism [103]. Endogenous CO is increased in G6PD-deficient neonates with hyperbilirubinemia. CO may have a role in promoting neuronal differentiation, as the CO-releasing molecule (CORM-A1) enhances neuronal differentiation in neuroblastomas [104]. The PPP pathway in neuroblastomas is upregulated by CO, including 6-phosphogluconate dehydrogenase (PGDH) from the oxidative branch of the PPP and transketolase (TKT) from the non-oxidative branch of the PPP. The concentration and activity of G6PD are also increased. Knockdown of G6PD reverses the effect of neuronal differentiation induced by CO [104]. This finding is indicative of a protective role of G6PD in the modulation of CO-induced neuronal development.

### 2.3. The Redox Role of G6PD in Pathophysiology

Altered G6PD status is implicated in many cellular pathophysiological processes and diseases, including hypoxia, inflammation, microbial infection, sepsis, pulmonary vessel dilation, diabetes, hypertension, kidney disease, and brain injuries [24,30,105,106,107,108,109,110,111,112,113,114,115,116,117,118,119,120,121,122,123,124,125,126]. The PPP and glutathione-associated metabolic pathways are major antioxidant defense systems in cells. The regulation of these enzymes profoundly affects the development and clinical outcome of diseases.

One of the pro-inflammatory conditions leading to vascular injuries is hyperglycemia. The pro-inflammatory cytokine IL-1β primes high glucose-induced vascular inflammation. In human aortic smooth muscle cells (HASMC), surplus glucose uptake can be activated by IL-1β [105]. Upregulation of the glucose transporter, GLUT-1 or downregulation of mitochondrial respiration alone is insufficient for stimulating the inflammatory response. IL-1β activates the PPP, where excess glucose reroutes to this pathway. This in turn overactivates NOX, which produces superoxide and its reaction with neighboring molecules such as NO, resulting in the production of free radicals that stimulate a downstream inflammatory signaling pathway that leads to endothelial dysfunction [105]. Chronic inflammation in adipose tissue is implicated in insulin resistance found in obesity [117]. Downregulation of G6PD, 6-phosphogluconate dehydrogenase (6PGD) and glutathione-S-transferase (GST) is found in the liver of aged and streptozotocin-induced diabetic rats [106]. The antioxidant, SMe1EC2, increases the G6PD activity but not 6PGD and GST in diabetic rats. SMe1EC2 can also enhance G6PD activity in the lung and heart of aged diabetic rats. These findings suggest that diabetes-induced glucotoxicity can be affected by modulating the activity of redox enzymes [24].

Diabetes is a condition that impairs the body’s ability to process blood glucose, including type 1, type 2, and gestational diabetes. G6PD deficiency could be a risk factor for diabetes. Impaired G6PD activity by high glucose concentrations in endothelial and kidney cells is associated with increased ROS production and decreased cell survival [127]. A decrease in G6PD expression and activity induced by ubiquitination and an increase in ROS in podocytes occurs at high glucose concentrations [128]. Hyperglycemia in obese mice results in increased oxidative stress in vascular endothelial cells and causes cardiovascular complications [129]. Smaller islets and impaired glucose tolerance are observed in G6PD-deficient mice [127]. Abnormal G6PD status mediates insulin resistance through oxidative stress in adipose tissue found in obese mice [118,119]. Upregulation of G6PD occurs in pancreatic β-cells in diabetic murine models [120]. Bone marrow transplantation from G6PD-deficient mice to wild-type mice reduces obesity-induced inflammation in adipose tissue and improves insulin resistance [107]. Overexpression of G6PD enhances ROS production and prooxidant enzymes, including iNOS and NOX [120].

ROS accumulation and β-cell apoptosis are indicative of the development of type 2 diabetes [130,131]. In high-fat-diet (HFD)-induced obesity, G6PD-deficient mice have decreased insulin and Homeostatic Model Assessment for Insulin Resistance (HOMA-IR) index [107]. These G6PD-deficient mice exhibit glucose and insulin tolerance as well as reduced insulin signaling, suggesting that G6PD deficiency is associated with an improvement in insulin resistance. Downregulation of NOX and upregulation of antioxidant genes, including catalase and glutathione peroxidase (GPx), are observed in G6PD-deficient mice. Although HFD-induced adiposity and fatty liver are not alleviated, the pro-inflammatory macrophages and cytokines are reduced in the G6PD-deficient mice. G6PD-deficient macrophages have decreased phosphorylation of MAPK, nuclear translocation of NF-κB, pro-inflammatory cytokines, and ROS accumulation. These events lead to enhanced insulin sensitivity in G6PD-deficient adipocytes and hepatocytes. Aberrant G6PD status in lipid-overload hepatocytes is associated with impaired pro-inflammatory cytokines via NF-κB and oxidative stress [117]. However, in some diseases with highly inflammasome activation, such as systemic lupus erythematosus, G6PD is upregulated (from array data GDS4185).

Microbial infection in G6PD-deficient patients is mainly related to hemolytic anemia caused by plasmodia or viruses [132,133,134,135]. Lack of G6PD activity is a risk factor for neonatal sepsis in males [108]. Sepsis-induced systemic inflammation and direct pulmonary injury cause acute lung injury (ALI) [136]. In airway epithelial cells (AECs), ALI can induce G6PD activity with the concomitant increase of ROS, nitrotyrosine, and NOX2 [110]. A G6PD inhibitor 6-AN suppresses airway inflammation in AECs induced by LPS and the ROS derived from NOX2, as well as increasing glutathione reductase activity. G6PD-knockdown A549 lung carcinoma cells are sensitive to staphylococcal infection, leading to apoptosis and ROS accumulation [109]. G6PD-deficient cells are susceptible to corona virus infection through inflammation and NF-κB signaling [111,112,113]. G6PD-deficient monocytes are more sensitive to infection by the dengue virus and have redox imbalance compared to matched control monocytes [115,116]. G6PD is highly expressed in human liver infected by the Hepatitis B virus (HBV) and HBV-associated cancer. G6PD activity can be stimulated by Hepatitis B virus X protein (HBx) mediated by the activation of the transcription factor, Nrf2 [30]. Low G6PD activity in children following acute hepatitis infection may cause high morbidity [114]. Mild hepatic encephalopathy (MHE) is a hallmark of chronic liver failure (CLF). The upregulation of G6PD and nNOS in MHE suggests that there is a role for NO in its pathogenesis [123]. Increased level of nNOS and NO is associated with increased activity of NADPH diaphorase in the cerebellum of CLF rats, where overactivation of G6PD is observed [123].

NADPH oxidation can regulate vascular muscle relaxation [137]. Dimerization of the 1α form of protein kinase G (PKG1α) induced by thiol oxidation contributes to the relaxation of isolated endothelium-removed bovine pulmonary arteries (BPA) to peroxide and responses to hypoxia [122]. G6PD inhibitors 6-AN and epiandrosterone are associated with enhanced PKG1α. An siRNA against G6PD increases PKG1α dimerization in BPA. Hence, reduced G6PD activity is associated with vasodilation, which may be beneficial in ameliorating pulmonary hypertension [121]. In addition, hypoxia activates G6PD and causes proliferation of the pulmonary artery smooth muscle (PASM) cell by increasing Sp1 and hypoxia-inducible factor 1α (HIF-1α), which synthesize less contractile (myocardin and SM22α) and more proliferative (cyclin A and phospho-histone H3) proteins. Consequently, G6PD overactivation contributes to remodeling of pulmonary arterial and development of pulmonary hypertension [138].

## 3. The Role of G6PD in Cell Growth and Development

G6PD is an archetypical housekeeping enzyme for maintaining growth and development. Diminished G6PD activity or a dysfunctional PPP prevents normal cell proliferation [52,139,140,141] as well as embryonic and organismal development [3,4,5,6,7,8,142]. Aberrant activation of the PPP or G6PD is associated with tumorigenesis [21,23,25,27,29,32,42,44,45,47,49,52,53,54,57,58,141,143,144,145,146,147,148,149]. Rapidly growing cancer cells have evolved myriad mechanisms to activate G6PD for supporting the cellular requirements for NADPH production and fatty acid and nucleic acid synthesis. For example, activation of pro-oncogenic pathways enhances G6PD activity, including Ras, Src, and PI3K/Akt [16,37,150,151]. NAD-dependent deacetylase Sirtuin 2, encoded by the SIRT2 gene, promotes NADPH production and leukemia cell growth through deacetylating G6PD [57]. Heat shock protein 27 (HSP27 or HSPB1) enhances the binding between G6PD and SIRT2, leading to deacetylation and activation of G6PD. HSPB1 activates G6PD through SIRT2 to sustain cellular NADPH and pentose production in glioma cells [152]. SIRT5 desuccinylates and deglutarylates isocitrate dehydrogenase 2 (IDH2) and G6PD, respectively, and thus activates both NADPH-producing enzymes and confers resistance to oxidative stress [153].

Signaling pathways governing cancer cell survival associated with G6PD status are discussed in this review, including signal transducers and activators of transcription (STAT), Wnt/β-catenin, AMP-activated protein kinase (AMPK), p21-activated kinases (PAK), as well as others listed in Table 1 and Table 2.

### 3.1. Cyclin and STAT3/5

Signal transducer and activator of transcription (STAT) proteins are transcription factors involved in immunity and cellular proliferation, apoptosis, and differentiation. Misregulation of STATs causes cellular transformation and tumorigenesis by compromising immune surveillance. STAT proteins are constitutively active and enhance the expression of pro-survival and pro-growth genes [156]. Although they do not directly contribute to the regulation of DNA repair and the cell cycle checkpoint, they facilitate oncogenesis through their close relationship with apoptosis, angiogenesis, and the growth factor signaling pathways [157].

In melanoma cells, STAT3 and STAT5 are highly activated. Knockdown of G6PD in melanoma cell lines leads to the reduction of phosphorylated STAT5 [157]. Overexpression of G6PD in human melanoma cells enhances phosphorylated STAT5. Likewise, phosphorylated STAT3 is modulated by G6PD status [157]. These findings indicate that G6PD has an important role in oncogenesis mediated by STAT3 and STAT5. However, the detailed mechanism between G6PD status and STAT3/5 activity remains unclear. Using a melanoma cell line xenograft nude mouse model, mice injected with wild-type G6PD cells display faster tumor formation and larger tumor size than those mice with G6PD-deficient cells [158]. Tumors of wild-type G6PD cells are more aggressively malignant compared to G6PD-deficient cells. These observations suggest that G6PD status is vital to melanoma proliferation and differentiation.

G6PD status in melanoma cells is positively correlated with the expression of the cell cycle proteins, including cyclin D1 and cyclin E [158]. S100A4, a calcium binding protein that binds to p53, is involved in cancer growth, invasion and metastasis [159]. As both proteins are elevated in melanoma cells, it has been proposed that G6PD modulates p53 activity, thereby affecting melanoma cell growth and metastases through its influence on S100A4 [157]. G6PD status is also correlated with apoptosis inhibitory factors. In G6PD-deficient melanoma cells, the expression of Bcl-2 and Bcl-xL is significantly reduced. Fas, a death domain-containing protein regulating programmed cell death, is highly expressed in G6PD-deficient melanoma cells [160]. The protein expression of the STAT3/5 ratio and the phosphorylated STAT3/5 ratio are decreased in G6PD-deficient melanoma cells. Lack of G6PD in mice melanoma cells enhances apoptosis by upregulating Fas and downregulating Bcl-2 and Bcl-xL [158]. Taken together, G6PD status is linked to cell proliferation and cellular malignancy, possibly through the STAT3/5 ratio, in mice melanoma cells.

### 3.2. ID1/c-Myc/Wnt/β-Catenin

The potential oncogene ID1 is found in several cancers in humans, including breast, kidney, pancreas, and prostate cancers [161,162,163]. ID1, an inhibitor of differentiation and DNA binding-1, is a member of the helix–loop–helix of transcription factors. Cellular ID1 is involved in delaying replicative senescence, inhibition of differentiation, enhancement of proliferation, invasion, immortalization, and metastases [164]. ID1 confers chemotherapy and radiotherapy resistance in breast, colorectal, esophageal, lung, and pancreatic cancers [165,166]. Elevated ID1 is correlated with a poor clinical outcome which includes shorter survival or resistance to therapies in breast, cervical, and non-small cell lung cancers [167,168,169]. Knockdown of ID1 in oxaliplatin-resistant hepatocellular carcinoma (HCC) cells reduces proliferation and induces apoptosis [33]. Consistent with the in vitro findings, silencing ID1 reduces the size and growth of tumor xenografts in nude mice. These tumors have a lower level of proliferation and a higher level of apoptosis. Silencing ID1 in HCC cells results in a decrease of G6PD activity, NADPH, and an increase in ROS. Transfection of G6PD into ID1-knockdown HCC cells reverses these findings and induces oxaliplatin resistance [33]. These results suggest that the malignancy and chemoresistance of HCC cells induced by ID1 are mediated by the activation of the PPP via G6PD.

The fact that ID1 silencing in HCC cells suppresses G6PD promoter activity facilitates the identification of c-Myc binding sites in the G6PD promoter sequence. The oncogene c-Myc is essential for the regulation of tumor cell cycle progression and metabolic adaptation. c-Myc regulates many genes that are involved in energy and glucose metabolism [170,171,172]. Transfection of c-Myc rescues the deficiency in ID1-knockdown in HCC cells. Pathway analysis indicates that ID1 activates c-Myc through Wnt/β-catenin. Activation of c-Myc through Wnt/β-catenin by ID1 is followed by the transcription of G6PD, thereby switching on the PPP, and consequently conferring HCC cells with chemoresistance to oxaliplatin [161].

### 3.3. AMPK

AMP-activated protein kinase (AMPK), ubiquitous in eukaryotes, is a heterotrimeric protein complex. It contains a protein kinase domain (α subunit), a glycogen binding domain (β subunit), and four cystathionine-β-synthase domains (γ subunit) [173]. A main function of AMPK is the monitoring of intracellular ATP fluctuations and the balancing of ATP levels by phosphorylation of downstream substrates. AMPK regulates cell growth and reprograms metabolism through transcription and by interacting with metabolic enzymes, including acetyl-CoA carboxylase (ACC), HMG-CoA reductase, and G6PD [174].

Reprogramming of energy metabolism is closely linked to metastases in tumor cells [175,176]. During metastases, reprogramming of glycolysis and the acidic environment enhance angiogenesis [177]. Increased carbon flux through the PPP enhances tumor cell malignancy and aggressiveness [178]. Attachment to the extracellular matrix (ECM) is critical for growth and differentiation of normal epithelial cells [179]. Upon detachment from the ECM, cells undergo apoptosis, known as anoikis [180]. Cancer cells need to adapt to the absence of the ECM in the circulation during metastases. Such a phenomenon is known as anchorage-independent growth. During this process, multiple survival-related cellular and molecular changes confer anoikis resistance in cancer cells.

Anchorage-independent growth is found in human breast cancer cells [51]. Compared to adhered breast cancer cells, detached cells display elevated ROS. Suppression of G6PD further increases the ROS level, suggesting that G6PD regulates intracellular ROS. The detached breast cancer cells compared to the adhered breast cancer cells have reduced glucose uptake, lactic acid, and ATP, indicating a reduction in glycolysis. Fatty acid oxidation (FAO) enzymes, including phospho-ACC and palmitoyl-transferase-1, as well as G6PD are upregulated in the detached cells. Inhibition with a newly synthesized flavonoid GL-V9 shows that FAO, not glycolysis, is the main source of ATP upon detachment. These findings suggest that during anoikis, detached cells shift glycolysis to the PPP in order to maintain redox homeostasis, while fatty acid oxidation is enhanced to support ATP. GL-V9 inhibits anchorage-independent growth through an increase in ROS. The mechanism involves the disruption of the balance between the PPP and FAO and the induction of glycolipid reprogramming. Activation of AMPK increases p-ACC and CPT1A. GL-V9 downregulates G6PD mRNA and its protein level through AMPK. Taken together, AMPK is activated by a flavonoid, causing a decrease in the PPP and FAO reprogramming and an increase in ROS. This results in the inhibition of anchorage-independent growth in breast cancer cells [51].

### 3.4. PAK4/Mdm2/E3/p53

The tumor suppressor p53 is a highly mutated gene in human cancers. p53 interacts with G6PD and regulates its function by preventing G6PD dimerization. Mutants of p53 in tumors lack G6PD-inhibitory activity [48]. Maintaining p53 activation is important for regulating cellular glucose consumption and biosynthesis via G6PD modulation. This suggests that other G6PD-interacting proteins can be used in cancers with p53 mutations. Members of the p21-activated kinases (PAKs) are serine/threonine protein kinases. The role of PAKs includes cell survival, cytoskeletal reorganization, gene transcription, and cell transformation. Overexpression of PAKs is commonly found in cancers [181,182,183,184]. The group II PAK4 is associated with tumorigenesis and progression. Although PAK1 is a regulator of glucose metabolism [185], it is not known if glucose metabolism is regulated by PAK4 in tumorigenesis.

PAK4 promotes lipid biosynthesis in colon cancer cells. Overexpression of PAK4 in colon cancer cells enhances glucose and NADPH production [186]. Elevation of G6PD activity is associated with PAK4 overexpression, while PAK4 knockdown reduces G6PD activity. DHEA decreases glucose consumption and NADPH. These findings indicate that PAK4-induced glucose consumption and NADPH production is mediated by enhanced G6PD activity. The close relationship between PAK4 and G6PD is supported by the fact that PAK4 binds to G6PD and the complex is co-localized in the cytoplasm. p53 is a downstream protein of PAK4 [48,187]. In vitro translated p53 binds to PAK4, suggesting that p53 interacts with PAK4. Silencing of PAK4 enhances p53 protein expression, indicating that PAK4 causes p53 degradation.

The ubiquitin proteasome pathway modulates p53 degradation [188]. PAK4 knockdown impairs p53 ubiquitination. Murine double minute 2 (Mdm2), a p53 antagonist, suppresses p53 activity by two mechanisms: inhibition of p53 transcriptional activity by direct binding and enhancement of p53 degradation through the Mdm2 component, E3 ligase [189]. PAK4 interacts with Mdm2, while PAK4 knockdown reduces the level of Mdm2. PAK4 facilitates the binding of Mdm2 and p53. These findings indicate that the enhanced interaction of Mdm2 and p53 caused by PAK4 can promote ubiquitin-mediated p53 degradation. In the absence of p53, PAK4 fails to affect glucose consumption and NADPH. PAK4 status in colon tissue from patients with colon cancer is positively correlated with the level of G6PD. This is in accord with the fact that PAK4 upregulates G6PD activity. Histological scoring shows that elevated PAK4 and G6PD is significantly linked to poor pathological tumor–node metastases (pTNM) [186].

## 4. The Role of G6PD in Cell Death

Dying cells engage in a reversible process until a first irreversible phase is trespassed, such as caspase activation, complete permeabilization of the mitochondrial outer membrane or exposure of phosphatidylserine (PS) sending the ”eat me” signal to neighboring cells [190]. The concept of the restriction point concerning cell death has yet to be specifically defined, and different types of cell death may occur at the same time. There are several types of cell death, such as regulated cell death (RCD), accidental cell death, and necroptosis, depending on different characteristics and criteria. Cells can readily switch from one type of cell death to another [191].

Based on the distinct cellular morphology, there are three major types of cell death, including type I (apoptosis), type II (autophagy), and type III (necrosis) [191,192]. Downregulation of G6PD impairs most cellular functions, especially regarding cell survival [1]. The major function of G6PD is the maintenance of cellular redox homeostasis by regenerating NADPH. A deficiency of G6PD makes cells more susceptible to stress by increasing oxidative damage [193]. G6PD-depleted embryos show more oxidative damage after the establishment of the blood circulation [3]. These studies indicate the importance of the antioxidant function of G6PD in growth and development [194].

### 4.1. Apoptosis

Apoptosis (extrinsic apoptosis) is the most common cell death caused by G6PD inhibition. G6PD deficiency enhances cellular oxidative stress and apoptosis that can be inhibited by free radical scavengers [195]. In erythrocytes, G6PD is the predominant enzyme for the production of NADPH against oxidative damage. Erythrocyte suicide, known as eryptosis, causes cell shrinkage, membrane blebbing, protease activation, and phosphatidylserine migration to the outer membrane leaflet [196,197]. In nucleated cells, G6PD inhibition induces apoptosis and suppresses cellular proliferation [127,198], especially in cells undergoing oxidative stress [4,199]. Increased glucose levels impair G6PD activity and result in apoptosis in kidney podocytes mediated by ubiquitination of G6PD at K366 and K403 [128]. The Von Hippel–Lindau (VHL) protein, a subunit of E3 ubiquitin ligase, binds to G6PD and undergoes ubiquitination in kidney podocytes. The ubiquitinated G6PD protein is degraded by the ubiquitin proteasome pathway, and cellular redox homeostasis becomes unbalanced by the increased oxidative stress [128].

### 4.2. Autophagy

Autophagy is a different programmed cell death characterized by large-scale vacuolization of the cytoplasm in a caspase-independent manner [200]. The autophagy–lysosomal pathway is a major pathway for removing damaged macromolecules and organelles [201]. Autophagic pathways can be stimulated by a number of events, such as exposure to pathogens and oxidative stress. Autophagy is modulated by redox homeostasis and glucose metabolism [202]. G6PD status can affect autophagy [26]. G6PD inhibition by polydatin induces endoplasmic reticulum stress and deregulates autophagy flux in cancer cells. Autophagosome formation is increased by polydatin. G6PD inhibition synergistically increases the cytotoxic effect of lapatinib in cancer cells, which can be abolished upon autophagy inhibition [26].

### 4.3. Necrosis and NETotic Cell Death

Necrosis is a premature cell death induced by injuries, including toxins, trauma or infections. Cells undergoing necrosis show swelling, cell membrane rupture, and expulsion of cell contents to nearby cells [203]. G6PD deficiency predisposes cells to the risk of viral and bacterial infections [109,111,116,204] followed by necrotic cell death. Pretreatment with antioxidant N-acetylcysteine (NAC) confers resistance to infection of G6PD-deficient cells [204]. NETotic cell death is another form of cell death in neutrophils by producing neutrophil extracellular traps (NETs). It is considered as regulated form of necrosis [205]. The function of NETs is to trap bacteria and kill them by protruding filaments consisting of fragmented chromatin and antimicrobial peptides [206]. NETotic cell death is regulated by NOX activity [207]. Severe G6PD deficiency increases the susceptibility to infection by the absence of NETotic cell death [208].

## 5. Strategies to Suppress G6PD

The modulation of G6PD status can be achieved by biochemical inhibitors or molecular technologies. These approaches are discussed in this section as well as listed in Table 3.

### 5.1. Biochemical Inhibitors

#### 5.1.1. Dehydroepiandrosterone

Dehydroepiandrosterone (DHEA) is an adrenal steroid whose biological role is not fully understood. G6PD can be inhibited by 17- and 20-ketosteroids [216]. DHEA is a potent noncompetitive inhibitor of mammalian G6PD. It decreases cellular NADPH levels and NADPH-dependent ROS production. The detailed mechanism of noncompetitive inhibition is with respect to both G6P and NADP+, where DHEA binds to the enzyme–coenzyme–substrate ternary complex [209]. DHEA has been widely used as a G6PD inhibitor to suppress the PPP in cancer cells [20,217].

#### 5.1.2. 6-Aminonicotinamide

Structurally similar to NADP, 6-Aminonicotinamide (6-AN) is an inhibitor of 6-phosphogluconate dehydrogenase. 6-AN is a competitive inhibitor of G6PD [218]. 6-AN also inhibits oxidoreductase and, in turn, the malic enzyme reaction [219].

#### 5.1.3. Polydatin

Polydatin (3,4′,5-trihydroxystilbene-3-β-d-glucoside (PD)), also named piceid, is a natural monocrystalline compound found in Polygonum cuspidatum (Polygonaceae) and other plants, such as grapes and peanuts [220]. PD is a glucoside of resveratrol. It has several biological effects, such as induction of apoptosis in cancer cells. PD inhibits G6PD activity and stimulates the generation of cellular ROS by increasing endoplasmic reticulum stress [210]. PD causes cell cycle arrest in the S phase, inducing about 50% apoptosis and inhibiting about 60% invasion. Currently, there is no specific G6PD or PPP inhibitors available in clinical trial. A phase II clinical trial shows PD is well tolerated in humans. It also limits cancer growth and metastatic spread in mice models of oral cancer [210].

#### 5.1.4. Zoledronic Acid

Zoledronic acid (ZA), also known as zoledronate, is currently a standard medication used to treat bone diseases, such as bone metastases and osteoporosis. ZA inhibits cell proliferation by decreasing the expression of G6PD in bladder cancer cells [211]. The stability of TAp73, the activator of G6PD, is decreased in ZA-treated bladder cancer cells through inhibition of Ras activity. This suggests that ZA can inhibit TAp73 stability and decrease G6PD activity via blocking Ras signaling in bladder cancer cells.

### 5.2. Noncoding RNA Regulation

#### 5.2.1. Small Interfering RNA

Small interfering RNA (siRNA) is a class of double-stranded RNA molecules with 20–25 base pairs in length. It mediates the interference of gene expression by its complementary nucleotide sequences to degrade mRNA. Due to the polymorphism of target genes, the specific sequence complementary to target genes needs to be carefully considered. Many studies on G6PD use siRNA to knockdown G6PD and investigate its role in cell lines. The human G6PD gene has more than 400 biochemical variants worldwide [221]. In this regard, the genetic polymorphism of G6PD needs to be considered for knocking down G6PD expression.

#### 5.2.2. Long Noncoding RNA

The genome sequences which do not encode proteins with lengths exceeding 200 nucleotides are usually transcribed into long noncoding RNA (lncRNA). LncRNAs play an important role in disease progression. Growth arrest-specific transcript 5 (GAS5), a lncRNA, has been identified in the dysregulation of the cell cycle in several cancers [222,223,224]. Melanoma samples from patients show significantly reduced GAS5 expression in advanced disease, such as larger tumor size and a high incidence of metastases [213]. GAS5 knockdown increases melanoma cell proliferation by inducing Cyclin D1, CDK4, and p27 expression and inhibiting apoptosis via increasing Bcl-2 expression. The use of RNA co-immunoprecipitation reveals that GAS5 interacts with G6PD. This results in inhibition of G6PD and NOX activity leading to a decrease in superoxide and NADP+. These data suggest that GAS5 could regulate cellular redox balance by directly interacting with G6PD.

#### 5.2.3. MicroRNA

MicroRNA (miRNA) are small noncoding RNA molecules that inhibit gene expression via base-pairing with complementary sequences within mRNA molecules. MiR-1, which belongs to the miR-1/206 family, is involved in heart diseases such as myocardial hypertrophy and infarction [225,226]. High concentrations of glucose lead to significant upregulation of miR-1 and miR-206 in cardiomyocytes. This results in an acceleration of glucose-mediated apoptosis [214]. In cardiovascular diseases, the upregulation of miR-1 increases cellular oxidative stress by downregulating superoxide dismutase 1 and G6PD protein expression [227]. miR-1 is involved in the regulation of G6PD expression through targeting the 3′ UTR sequence [41,227,228]. This indicates that the inhibition of G6PD by high glucose concentrations may be due to the upregulation of miR-1. Another miRNA candidate of G6PD inhibition is miR-122, which is the liver-specific miRNA. MiR-122 decreases G6PD expression by directly interacting with its 3′UTR as determined by the luciferase reporter assay. Ectopic expression of miR-122 decreases G6PD expression in hepatocellular carcinoma cells and HepG2 cells [55]. These data indicate that miR-1 and miR-122 regulate the PPP by inhibiting G6PD.

### 5.3. Other Approaches

#### 5.3.1. TERT Regulation

Telomerase is a ribonucleoprotein with three components, including a human telomerase RNA subunit, a telomerase-associated protein, and a human telomerase reverse transcriptase (hTERT). Its main function is to insert a telomere repeat sequence at the 3′ end of telomeres [215]. The maintenance of telomeres supports long-term cellular growth [229]. Active telomerase is commonly observed in stem cells and human cancers [230]. In glioma cells, costunolide treatment inhibits hTERT activity and induces cell apoptosis by an ROS-dependent pathway. hTERT inhibition by costunolide, or by molecular technologies, such as siRNA or dominant-negative hTERT, attenuates G6PD expression [215]. This indicates that there is a possible role for G6PD in the Nrf2-TERT loop for maintaining the oxidative defense responses in astrocytes or glial cells.

#### 5.3.2. Protein-Protein Interactions

The Bcl-2 associated athanogene 3 (BAG3) protein is involved in several cellular functions, such as autophagy, cell cycle regulation, cellular development, and pathogen replication [231,232]. In addition to the interaction with the ATPase domain of Hsc/Hsp70 family, it also contains multiple protein-binding motifs interacting with chaperons. In hepatocellular carcinoma cell models, BAG3 protein directly interacts with G6PD by inhibiting G6PD dimerization and activity. Such an interaction decreases the PPP flux and cell growth without altering cellular NADPH. BAG3 protein directly binds to G6PD to exert the tumor suppressor-like function in HCCs [154].

### 5.4. G6PD as the Basis for Therapeutic Approaches

Several G6PD inhibitors induce apoptosis in tumor cells. In noncoding RNA regulation, RNA-based therapeutics might be a reasonable approach in cancer therapy. Although there has been some success with preclinical studies utilizing RNA-based therapy, few have proceeded to clinical trials [233]. The challenges for RNA-based therapeutics include an off-target effect, target gene polymorphisms, and ineffective delivery systems to the site of interest [234].

6-AN is used for chemotherapy in various cancers, but the treatment is always associated with severe nerve damage [235]. Clinical trials with DHEA have been unsuccessful because of the need for high oral doses and the conversion of DHEA into other active forms [11]. Although PD and ZA have been used in clinical trials for the treatment of irritable bowel syndrome [234] and Paget’s disease of bone, respectively, the detail mechanism of G6PD inhibition of PD and ZA requires further elucidation.

Since G6PD is a biomarker and G6PD inhibition has potential as a therapy for cancer, a promising series of recently developed inhibitors could be designed according to their interaction. Using the backbone of the inhibitor to develop a novel and powerful G6PD inhibitor has become an important issue in cancer therapeutics. Computational methods have been used to analyze the binding structures and free energies of G6PD for designing potential DHEA derivatives [236]. This establishes valuable insights into detailed enzyme-inhibitor binding. Such in silico findings can provide a new understanding for the possible design of potent G6PD inhibitors for the treatment of diseases.

## 6. G6PD/PPP as an Anticancer Target

Tumorigenesis is a dynamic and complex process, in which each state of the tumor development is closely connected. A dynamic change in glucose metabolism is a feature of cancer cells. The Warburg effect is characterized by the fact that cancer cells favor glycolysis with increased lactate production even in the presence of oxygen rather than the oxidative phosphorylation pathway, which is the preference of most other cells of the body [237]. The metabolic switch is known as the Warburg effect and is responsible for promoting the synthesis of essential cellular components in rapidly-proliferating cancer cells. The metabolic switch affects cancer cells and other immune cells, such as macrophages, T lymphocytes, and myeloid-derived suppressor cells (MDSCs) [238]. Targeting metabolic switches or metabolism-regulated signaling pathways in tumor development can be a potential strategy for anticancer treatment, or in combination with immunotherapies.

The metabolic switch plays an important role in tumor growth, including immune escape, tumor progression, and resistance to chemotherapy. Activation of G6PD is the first line response against oxidative stress in cancer cells [239]. Cancer cells obtained from thoracic esophageal squamous cell carcinoma patients utilize the PPP in order to meet the need for rapid growth and for developing resistance to chemotherapy [38,240,241]. Oxidation of G6PD, post-translational modification or allosteric interactions are the direct result of inhibition by NADPH during acute stress-induced glucose rerouting [242,243]. This may explain why G6PD is important in redox-sensitive metabolism and is required in rapidly proliferating cells. In bladder cancer cell metabolism, increased expression of G6PD and fatty acid synthase are observed. This favors increased metabolism of glucose by the PPP and increased fatty-acid synthesis [37]. PPP hyperactivation may act procarcinogenically in bladder cancer cells [211]. Mitigation of the Ras–TAp73–G6PD pathway by ZA results in the inhibition of G6PD, leading to retardation of bladder cancer cell proliferation. This indicates that the Ras–TAp73–G6PD pathway may be the target for G6PD inhibition. Metabolic analysis of acute myeloid leukemia (AML) cells has revealed that the PPP acts as an important pro-survival pathway by increasing mTORC1 activity [31]. Overexpression of G6PD correlates with an adverse prognosis and has been pinpointed as a new biomarker in AML as determined by analysis of the cancer genome atlas AML database. G6PD inhibition induces in vitro and in vivo chemotherapeutic cytotoxicity in AML cells, demonstrating that high mTORC1 expression may be a target for G6PD inhibition. Unlike in cancer cell studies, the metabolic switch is also important in neuronal development [244]. Cdh1 is involved in regulating neuronal survival, especially in volatile anesthetic-induced neuronal apoptosis. Prolonged sevoflurane anesthesia significantly decreases Cdh1 and results in a glucose metabolism shift from the PPP to neuronal glycolysis, leading to higher susceptibility to oxidative stress in the brain. This indicates that Cdh1 may be a novel target for glucose metabolic reprogramming.

Mutations of FMS-like tyrosine kinase 3 (FLT3) are often observed in acute myeloid leukemia (AML). This is most likely why FLT3 inhibitors ultimately fail to achieve long-term remission. Ataxia telangiectasia mutated (ATM) is identified as the reason for resistance to FLT3 inhibitor therapy as found by a genome-wide RNAi-based screen. Inactivating ATM or its downstream effector G6PD sensitizes AML cells to FLT3 inhibitor-induced apoptosis [245]. These studies indicate that G6PD plays an important role in cancer metabolism. Its key role in cancer promotes cell proliferation and antioxidation. The use of a G6PD inhibitor increases cancer cell death. This could be a strategy for combining with other cancer therapies, such as radiotherapy, chemotherapy, and immunotherapy.

The upregulation of the PPP in cancer cells makes it a potential target for cancer therapy. A major characteristic of cancer cells in coordinating glucose utilization in response to cell physiology is the reprogramming of glucose metabolism. The rerouting of glucose to the PPP produces high levels of NADPH to counteract the ROS, while providing nucleotides for DNA synthesis. These activities confer resistance to elevated ROS and DNA damage. Increased PPP activity induces high levels of ROS. This is counteracted by an adaptive response that is found with the use of chemotherapeutic agents [26,32,246], radiation [247,248,249,250], and oxidative agents [28,239,251]. Drug-resistant cancer cell lines display increased G6PD activity and increased intracellular glutathione concentrations indicative of oxidative PPP. Enhanced non-oxidative PPP is linked to the resistance of DNA-damaging drugs, like 5-fluorouracil (5-FU) [252]. Elevated TKT is detected in colon cancer cells. Although increased PPP is a weapon used by drug-resistant cancer cells, modulation of the PPP status can serve as a strategy for sensitizing cancer cells to therapies. Doxorubicin, a member of the anthracyclines, is used in chemotherapy. It is metabolized by cytochrome p450 and causes cytotoxicity by producing ROS [253]. Reduced G6PD activity has been found in doxorubicin-resistant breast cancer cells. Stimulation of the PPP may sensitize cancer cells to doxorubicin. Treatment of breast cancer cell lines with l-arginine in combination with 5-FU enhances apoptosis and decreases metastases by targeting G6PD [36]. 6-AN, is cytotoxic in acute myeloid leukemia cells and sensitizes these cells to chemotherapy [31]. Inhibiting G6PD in breast cancer cells synergistically enhances the anti-HER2 tyrosine kinase inhibitor-induced cytotoxic effect [26].

Such metabolism-based therapeutic strategies might be a solution for impeding rapid growth and metastases as well as cancer cell heterogeneity in cancer stem cells [144,254,255]. The activity and localization of G6PD are regulated by several proteins, including STAT, ID1, p53, BAG3, PAK4, HGF, and AMPK [33,48,51,154,186]. Disrupting the interaction between G6PD and these proteins can inhibit the reprogramming of glucose toward the PPP and impair the biosynthesis of the building blocks of cancer cells associated with a decrease in intracellular NADPH. This can be another potential therapeutic strategy to thwart rapidly growing cancer cells.

## 7. Conclusions

The main function of G6PD is to provide sufficient reducing power to support growth and maintain redox homeostasis. Studies regarding G6PD deficiency have traditionally focused on red cell disorders. Current studies on nucleated cells reveal that G6PD participates in a variety of cellular processes via redox signaling. A close relationship exists between G6PD-derived NADPH and reactive species. In particular, NOX and NOS are two major NADPH-dependent enzymes that generate reactive species involved in cellular signaling. Altered redox homeostasis in G6PD-deficient cells leads to impaired antioxidant defenses and cellular signaling. G6PD deficiency is associated with a myriad of pathological events and diseases.

G6PD is critical in signaling for governing cell growth and cell death. Aberrant activation of G6PD is linked to tumorigenesis and malignancy in rapidly growing cancer cells. This raises the possibility of whether or not G6PD can serve as a therapeutic target concomitant with existing anticancer drug to tackle cancer resistance [256]. Approaches with chemical or molecular inhibitors of G6PD have been used widely. Novel methods of inhibiting the action of G6PD through modulating the metabolic switch, redox homeostasis, and protein–protein interactions are of great interest in thwarting rapid growth, metastasis, and heterogeneity of cancer cells.

## Figures and Tables

**Table 1:** The effects of upregulated glucose-6-phosphate dehydrogenase (G6PD) in cells.

Cancer Type	Effects of G6PD Activation	Mechanism	Reference
Breast	Enhanced proliferation and migration	Nrf2 upregulates Notch1 and HES-1(proliferation) via G6PD/HIF-1 and regulates EMT (migration)	[44]
	Increased glucose uptake and NADPH production	Histone deacetylase inhibitor reprograms metabolism by upregulating G6PD	[32]
	Elevated NADPH, Reduced ROS	Overexpression of Histone H3K36 methyltransferase (NSD2) methylates the promoters and upregulates hexokinase 2(HK2) and G6PD	[54]
Leukemia	Enhanced cell proliferation and colony formation, lipid synthesis, and NADPH level	Deacetylation of G6PD by SIRT2	[57]
Lung	Increased glucose flux through PPP, Enhanced tumor growth as well as production of nucleotide, lipid and reducing equivalents	Glycosylation (O-GlcNAcylation) of G6PD	[45]
Ovarian	Cancer progression and carcinogenicity	Exosomes	[145]
Renal	Increased cell proliferation, altered cell cycle, increased ROS production	Activation of NOX4 leads to increased p-STAT3 and CyclinD1	[146]
Glioma	Increased cell proliferation, reduced DNA damage	Hsp27 (HSPB1) promotes the interaction between G6PD and SirT2	[152]
Multiple cancers	Cell cycle progression and cell proliferation	Phosphorylation of G6PD by Polo-like kinase 1(Plk1)	[49]

**Table 2:** The effects of downregulated G6PD in cells.

Cancer Type	Effects of G6PD Deficiency	Mechanism	Reference
Bladder	Reduced cell viability and growth, increased apoptosis	Increased ROS accumulation. Suppression of AKT	[42]
Breast	Reduced cell proliferation, cell survival, increased ROS, decreased ribose (in combination with TKT deficiency)	Increased glycolytic flux and glutamine intake. Decreased lipid synthesis	[25]
	Increased autophagosome, impairment of autophagy flux, increased lapatinib-induced cytotoxicity	Induced endoplasmic reticulum stress	[26]
Cervical	Inhibition of viability, decreased migration and proliferation, abnormal cytoskeleton reorganization	Increased ROS induces apoptosis	[20]
	Reduced cell proliferation	miR-206 targets 3′UTR of G6PD	[40]
	Inhibition of proliferation, promotion of apoptosis, reduced xenograft tumor growth in nude mice	miR-1 suppresses G6PD activity	[41]
Colon	High NADP, inhibition of dihydrofolate reductase (DHFR), impairment of folate-mediated biosynthesis	Induced ME1 and IDH1 compensation	[43]
Colorectal	Decreased synthesis of ribose and NADPH	Acetylation of G6PD at catalytic site by aspirin	[18]
Hepatocellular	Suppressed PPP flux, DNA synthesis, and cell growth	Bcl-2 associated athanogene (BAG3) suppresses dimerization and activity of G6PD	[154]
	Reduced G6PD activity	miR-1, miR-122 repress G6PD expression	[55]
Leukemia	Increased cytotoxicity, sensitivity to chemotherapy	Overactivation of TORC1	[31]
Lung epithelial	Induction of apoptosis, generation of ROS	Activation of TRAIL, FAS, TNF-α receptors, caspase3/9 by phytol	[155]
Melanoma	Cell cycle arrest, blockade of cell proliferation	Downregulation of cyclin D1 and CDK4, upregulation of p53 and p21	[16]
Pituitary	Inhibition of growth, Reduction of NADPH, Reduction of glycolysis	Upregulation miR-1 inhibits G6PD	[19]
Multiple cancers	Reduction of ribonucleotide and GSH and cell proliferation	Phase 2 drug (RRx-001) downregulates G6PD	[56]

**Table 3:** Strategies of G6PD inhibition.

Status	Mechanism	Reference
High glucose	Ubiquitination and degradation of G6PD	[128]
G6PD inhibitors		
DHEA	Uncompetitive inhibitor of G6PD	[20,209]
6-AN	Competitive inhibitor of G6PD	[42]
Polydatin	Inhibition of G6PD activity	[210]
Zoledronic acid	Inhibition of G6PD activity	[211]
Metabolic switch	From PPP to glycolysis	[31,211,212]
Noncoding RNA regulation	Direct target to G6PD mRNA or target to G6PD mRNA’s 3′-UTR	[7,41,55,213,214]
TERT regulation	Inhibition of hTERT decrease G6PD expression	[215]
Protein-protein interaction	BAG3 directly interacts with G6PD	[48,154]
